# Risk Assessment of the Role of the Ecotones in the Transmission of Zoonotic Cutaneous Leishmaniasis in Central Tunisia

**DOI:** 10.3390/ijerph18179274

**Published:** 2021-09-02

**Authors:** Walid Barhoumi, Ifhem Chelbi, Wasfi Fares, Sami Zhioua, Mohamed Abbas, Mohamed Derbali, Marcelo Ramalho-Ortigao, Elyes Zhioua

**Affiliations:** 1 Laboratory of Vector Ecology, Pasteur Institute of Tunis, Tunis 1002, Tunisia; walidbarhoumi.ipt2009@yahoo.fr (W.B.); ifhemc2001@yahoo.fr (I.C.); fwasfi@yahoo.fr (W.F.); m.abbas9900@gmail.com (M.A.); derbali.hiba@gmail.com (M.D.); 2 Laboratory of Clinical Virology, Pasteur Institute of Tunis, Tunis 1002, Tunisia; 3 Laboratory of Bio-Informatics, Mathematic, Biostatistics, Pasteur Institute of Tunis, Tunis 1002, Tunisia; sami.zhioua2@gmail.com; 4 Department of Preventive Medicine and Biostatistics, Uniformed Services University, Bethesda, MD 20814, USA

**Keywords:** sand flies, rodents, ecotones, transmission dynamics, zoonotic cutaneous leishmaniasis

## Abstract

Zoonotic cutaneous leishmaniasis (ZCL), endemic in Central and Southern Tunisia, is caused by *Leishmania major* (Kinetoplastida: Trypanosomatidae), which is transmitted by the sand fly *Phlebotomus papatasi*. In Tunisia, the fat sand rat *Psammomys obesus* and the desert jird *Meriones shawi* are the principal reservoir hosts of *L. major*. The presence of the *P. papatasi* vector of the *L. major* etiologic agent of ZCL was assessed in the vicinity of villages in endemic areas of Central Tunisia. The study was performed from September through October 2019, a period corresponding to the main peak of activity of *P. papatasi*. Sand flies were collected from rodent burrows located at the ecotone level, which is the transition zone between the natural environment and human settlement. Sand flies were identified to species level and tested for the presence of *L. major* by PCR. Our entomological survey showed that *P. papatasi* is the most abundant sand fly species associated with rodent burrows, and this abundance is even higher in ecotones primarily occupied by *P. obesus* in comparison to ecotones occupied by *M. shawi*. Infections with *Leishmania major* were detected only in *P. papatasi*, with an overall minimum infection rate (MIR) of 2.64%. No significant difference was observed between the MIRs in ecotones of *P. obesus* and of *M. shawi*. Incidence of ZCL in the studied areas ranged from 200 to 700 cases per 100,000 inhabitants, with a mean incidence of 385.41 per 100,000. Higher ZCL incidence was identified in ecotones of *M. shawi* compared to ecotones of *P. obesus*. ZCL cases are positively correlated with the MIRs. Considering the short flight range of *P. papatasi*, increases in its densities associated with burrows of *P. obesus* or *M. shawi* at the ecotone level expand the overlap of infected vectors with communities and subsequently increase ZCL incidence. Therefore, control measures should target *P. papatasi* populations at the ecotones.

## 1. Introduction

In North Africa, zoonotic cutaneous leishmaniasis (ZCL) is caused by the parasite *Leishmania major* and is transmitted by infected bites of the sand fly species *Phlebotomus papatasi* [1,2]. In several ZCL endemic areas throughout North Africa, the fat sand rat *Psammomys obesus* and the desert jird *Meriones shawi* are the principal reservoir hosts of *L. major* [3,4,5,6,7,8]. Annually, thousands of ZCL cases are reported from Central and Southern Tunisia [9,10], with the governorate of Sidi Bouzid alone having an estimated annual ZCL incidence rate of 669.7 per 100,000 inhabitants [11]. Although ZCL is not fatal, the lesions produced may cause substantial disfigurement and severe distress to infected individuals with lifelong psychological and social consequences [12]. There is no ZCL vaccine available, and treatment is based primarily on chemotherapy. In addition, programs concerning vector and reservoir host control are currently absent for the prevention of this neglected tropical disease in Tunisia.

Roughly 63.4% of ZCL cases are reported from rural areas, which account for 71% of the total population of Central Tunisia [11]. Rural areas located in Central Tunisia are characterized by poor housing conditions where villages are near and often surrounded by biotopes associated with reservoir hosts of *L. major*. Two clearly identifiable ecotones exist separating housing developments from rodent burrows. One ecotone is principally occupied by *P. obesus* whose main food sources are Chenopodiaceae such as *Salsola tetrandra*, *Suaeda fruticosa*, and *Arthrocnemum glaucum* [13]. These chenopod fields represent the natural habitat of *P. obesus*; in addition, they are used as grazing field for livestock [13]. The other ecotone is occupied by *M. shawi* and consists of agricultural fields associated with the jujube tree, *Ziziphus zizyphus,* which provides shelter from predators while serving as a critical source of food in the form of the jujube fruit [8]. Because of its wide food diet, *M. shawi* has a more migratory behavior in comparison to *P. obesus* which is more sedentary. Each rodent species inhabits a different complex burrow system. The rodent burrows have moderate, stable temperatures and elevated humidity creating a suitable microclimate for the immature and adult stages of *P. papatasi* [8]. Adult female *P. papatasi* also utilize these rodent species as their primary blood meal source, and the rodent feces and plant debris that accumulate in these burrows are the main food source for sand fly larvae [8]. As a general assumption, adult sand flies in villages come from breeding and resting sites located in the surrounding land [14]. This transition zone between two adjacent ecological systems is defined as the ecotone [15]. From an epidemiological point of view, ecotones represent a specialized habitat as the interface between human settlements and natural ecosystems where humans come in contact with rodent reservoirs and vectors leading to emergence of zoonotic and vector-borne diseases. We hypothesized that *L. major* infection prevalence of *P. papatasi* associated with rodent burrows located at the ecotones is a key determinant of the force of infection in ZCL endemic foci.

## 2. Materials and Methods

### 2.1. Study Sites

**Figure 1:**
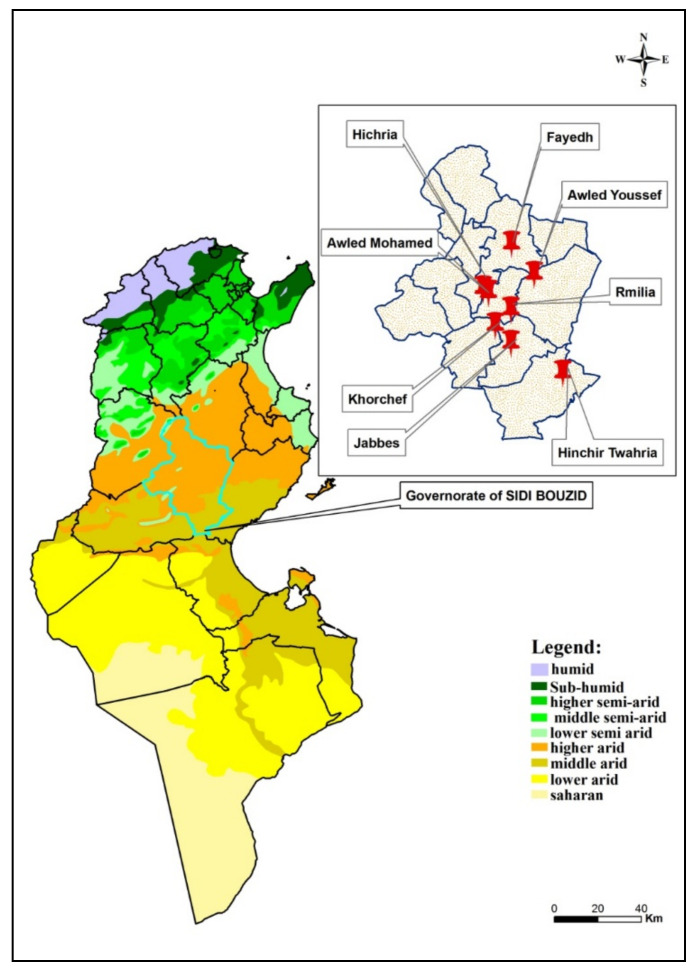
Bioclimatic map of Tunisia showing sampling sites in the governorate of Sidi Bouzid.

The study was performed in nine villages belonging to different delegations within the governorate of Sidi Bouzid, a highly endemic area with multiple foci of ZCL located in Central Tunisia [11] (Figure 1).

**Figure 2:**
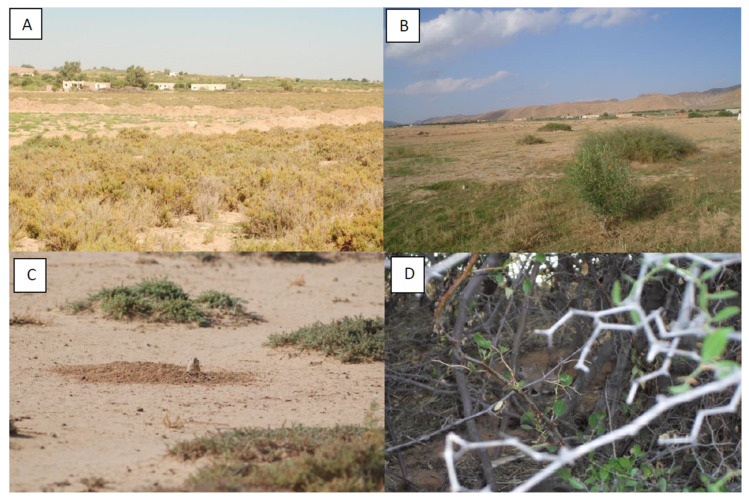
Ecotone of *Psammomys obesus* (**A**,**B**) and *Meriones shawi* (**C**,**D**).

Villages are frequently found surrounded by fields of chenopods (Figure 2A), or by agricultural fields harboring jujube trees (Figure 2B), which are the natural habitats of *P. obesus* (Figure 2C) and of *M. shawi* (Figure 2D), respectively. Therefore, study sites were either the ecotone of *P. obesus* or of *M. shawi*.

### 2.2. Sand Fly Trapping and Identification

The phenology of *P. papatasi* in Tunisia is characterized by two main peaks of activity: a small one in June and a second larger one in September–October [9]. Sand fly trappings were performed during the second peak in 2019. Sand flies were trapped using sticky traps placed overnight at the entrances of active rodent burrows at each site. Each trap consisted of 13 white sheet papers (20 cm × 20 cm) soaked in castor oil, yielding a total surface of 1 m^2^ (one paper trap per active burrow; paper traps were placed between two and ten meters apart, with a total of 50 to 200 paper traps per site). The total number of trap-nights was 19,986 (Table 1). Active rodent burrows were characterized by the presence of Chenopodiaceae fragments, feces and urine at their entries [6,7,8]. All trapped sand flies were individually identified according to morphological characters [16]. Collected unfed female sand flies were pooled based on collection date, with up to a maximum of 30 unfed females per pool, and stored at −80 °C until use.

### 2.3. Detection of Leishmania DNA in Female Sand Flies

Pools of unfed female sand flies (with up to 30 females for pool) were homogenized by hand for 2 min in 100 µL of phosphate-buffered saline (PBS), and an additional 100 µL of PBS was added to each pool for a final volume of 200 µL. The mixture was clarified by centrifugation at 6000× *g* for 2 min to be used for DNA extraction with Qiagen DNA Mini Kit (Qiagen). *L. major* DNA extracted previously from parasite culture was used as a positive control. Extracted DNA was screened for infections of *Leishmania* species by nested PCR of a partial region of ITS-rDNA gene as previously described [17,18]. The first amplification steps were performed using the Taq DNA recombinant polymerase kit (Invitrogen) in 50 µL reaction containing 5 µL 10X buffer, 3 µL MgCl2 (50 mM), 2 µL dNTP mix (10 mM), 1 µL of each reverse and forward primers IR1/IR2 (10 µM), 0.5 µL Taq DNA polymerase enzyme, and 10 µL of total extracted DNA. The nested PCR was carried out in 50 µL containing 2 µL of the first PCR step DNA product and 48 µL of mixture containing 5 µL 10X buffer, 3 µL MgCl2 (50 mM), 2 µL dNTP mix (10 mM), 1 µL of each reverse and forward internal primers ITS1F/ITS2R4 (10 µM), and 0.5 µL of Taq DNA polymerase (Invitrogen). Optimized cycling conditions for the first and second PCR step were performed as follows: (*i*) 94 °C for 3 min followed by 40 cycles of 94 °C for 60 s, 58 °C for 60 s and 72 °C for 90 s, followed by a final extension step (72 °C) for 10 min; (*ii*) nested PCR with 94 °C for 3 min followed by 5 cycles of 94 °C for 60 s, 55 °C for 60 s and 72 °C for 60 s, and 35 cycles of incubation at 94 °C, 59 °C and 72 °C for 60 s each. The extension step was continued for 10 min at 72 °C. Cross-contamination was monitored by negative controls for sample extraction and PCR solutions, for PCR test. Amplification products of the nested PCR were separated in 2% agarose gel stained with ethidium bromide and visualized under UV light illumination. Positive PCR products were directly sequenced to identify sand fly-associated *Leishmania* species.

### 2.4. DNA Sequencing and Phylogenetic Analysis

The 462bp nested PCR products were purified by the ExoSAP-IT method using Exonuclease-I and Shrimp Alkaline Phosphatase and sequenced in both directions using a Big Dye Terminator ready reaction cycle sequencing v3.1 kit (Applied Biosystems, Waltham, MA, USA) with forward and reverse nested PCR primers (ITS1F/ITS2R4) [19]. Resulting consensus sequences were deduced by aligning the respective forward and reverse sequences using CLUSTAL_W 1.4 implemented in MEGA v.5.22 [20]. In addition to the studied sequences, several *Leishmania* species sequences were selected from gene bank databases, including 1 *L. infantum* sequence, 1 *L. tropica* sequence, and 22 *L. major* sequences. Phylogenetic analysis was performed using the neighbor-joining analysis method and the kimura-2 model. The tree topology was supported by 1000 bootstrap replicates.

### 2.5. Statistical Methods

In this study, abundance was analyzed with contingency tables. The infection of *P. papatasi* with *L. major* was reported using the minimum infection rate (MIR), which was calculated as follows: ([number of positive pools/total number of tested sand flies] × 100) [21]. A Wilcoxon rank sum test was used to analyze the difference(s) in MIRs between biotopes. To analyze the difference between MIR medians among delegation, a Kruskal–Wallis rank sum test was used.

All data from clinically confirmed cases during the ZCL epidemiological season starting in July 2019 until June 2020 were obtained from the Regional Health Department of Sidi Bouzid. Numbers of ZCL cases were given by delegation. The ZCL incidence between ecotones was analyzed with a Wilcoxon rank sum test; *p*-values were significant at a value of 0.05. Relationships between factors were tested with a Pearson’s correlation. All tests were computed using R v. 3.6.0.

## 3. Results

A total of 885 male and 564 female sand flies were collected from all investigated sites. Overall, *P. papatasi* was the most abundant species (73.29%) followed by *Sergentomyia minuta* (18.70%), *Sergentomyia fallax* (5.72%), *P. longicuspis* (0.55%), *Sergentomyia christophersi* (0.55%), *Sergentomyia deryfussi* (0.06%), and *Sergentomyia antennata* (1.1%) (Table 2). The abundance of *P. papatasi* was significantly higher compared to the abundances of other sand fly species (Chi-squared = 626.38, df = 1, *p*-value < 0.001). The abundances of *P. papatasi* in the ecotones of *M. shawi* (256) and of *P. obesus* (806) were significantly different (Chi-squared = 553.02, df = 1, *p*-value < 0.001).

A total of 24 pools of female *P. papatasi* were screened for *Leishmania* infection by nested PCR. Eleven pools were found to be infected with *Leishmania* DNA. Only pools of *P. papatasi* were infected with *Leishmania* DNA. Thus, the overall minimum infection rate of *P. papatasi* with *Leishmania* DNA was 2.64% (11/417) (Table 3). The mean MIR (mean ± SE) in ecotones of *P. obesus* and of *M. shawi* were 3.46 ± 1.33 and 1.92 ± 1.92, respectively. Among ecotones, no significant difference was observed between the minimum infection rates (Wilcoxon rank sum test: W = 11.5, *p*-value = 0.2907). MIR medians were not significantly different among delegations (Kruskal–Wallis rank sum test: Kruskal–Wallis chi-squared = 1.9512, df = 3, *p*-value = 0.5826).

The incidence of ZCL varied from 200 to 700 cases per 100,000 inhabitants among studied sites (Table 4). The mean ZCL incidence among delegation was 385.41 ± 77.29. The mean ZCL incidences in the ecotones of *M. shawi* and of *P. obesus* biotopes were 504.76 ± 123.72 and 266.06 ± 19.93, respectively. ZCL incidence medians differed significantly between ecotones (Wilcoxon rank sum test: W = 9, *p*-value = 0.04953).

**Figure 3:**
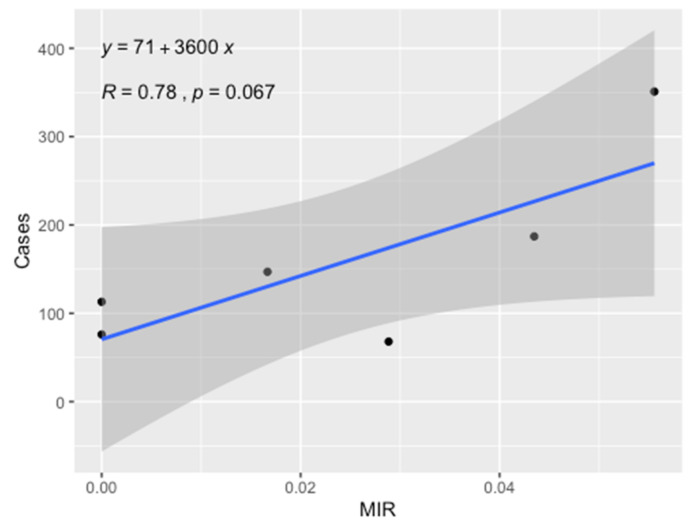
Correlation between MIRs and ZCL cases.

A positive correlation (0.78) between the MIR and the number of clinical cases was observed, with results approaching significance (Pearson’s correlation: t = 2.4986, df = 4, *p*-value = 0.06687, Figure 3). 

### Leishmania Sequencing and Phylogenetic Analysis

**Figure 4:**
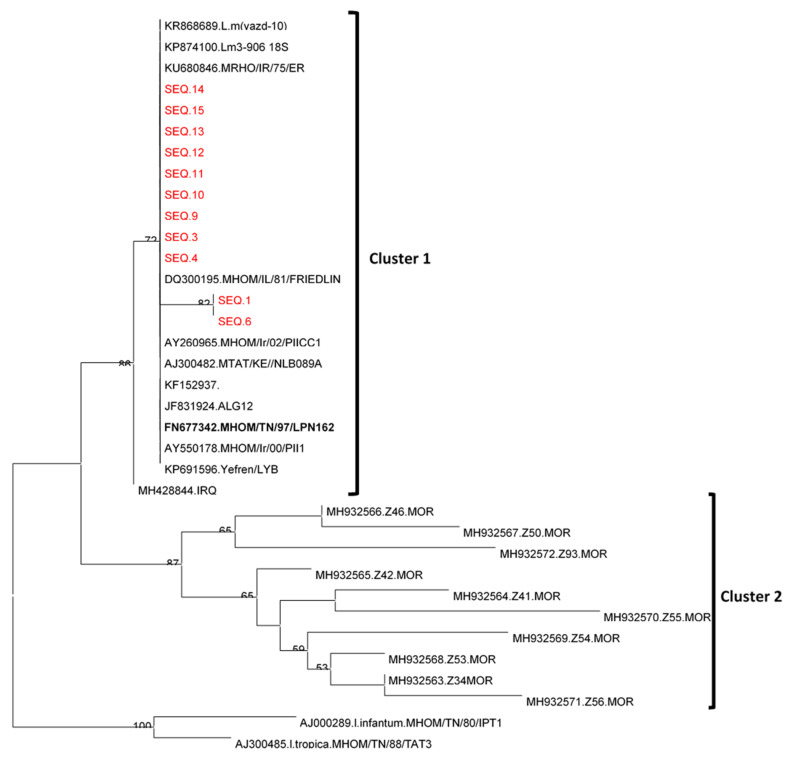
Phylogenetic tree based on partial *Leishmania* ITS-rDNA 5.8 s sequences. The tree includes 35 *Leishmania* ITS-rDNA 5.8 s sequences distributed as follows: 11 *L. major* sequences reported in this study, 22 previously published *L. major* sequences, and two representative sequences of *L. infantum* and *L. tropica* species. The phylogenetic tree was generated from the alignment of a 277 bp fragment of the ITS-rDNA obtained from the trimming of the sequences used, followed by maximum likelihood model analysis using Jukes–Cantor parameter. The tree topology was supported by 1000 bootstrap replicates, and bootstrap values lower than 50 were hidden.

Blast analysis indicated that identified sequences were closely related to the *L. major* reference sequence isolated from a Tunisian patient in 1980 (FN677342.L.major.MHOM/TN/97/LPN162). All *L. major* sequences clustered together with other *L. major* strains from different North African and Middle Eastern countries, separately from *L. tropica* and *L. infantum,* which appear in two different clusters (Figure 4). Tree topology showed that *L. major* sequences were divided into two clusters: Cluster 1 included sequences from different North African and Middle Eastern countries, while Cluster 2 was represented only by sequences from Morocco. These two phylogenetic branches including Cluster 1 and Cluster 2 were supported by high bootstrap values (86%) (Figure 4). Phylogenetic analysis revealed two sub-clusters within the same cluster, one composed of nine *L. major* sequences grouped together with the reference Tunisian isolate FN677342.MHOM/TN/97/LPN162 and a second one made of two *Leishmania* sequences grouped together. These two sub-clusters differed only by a single nucleotide, thus presenting a low nucleotide divergence rate of 0.08%.

## 4. Discussion

Sand flies were collected in sites located at the edge of rural villages where cultivated plains overlap with fields of chenopods or jujube trees, the natural habitat of the rodent reservoirs forming an ecotone, which is the interface between human settlements and natural ecosystems [15]. Previous study performed in Southern Tunisia showed that cases of *L. major* are clustered at the margin of some villages where the gerbil reservoir hosts (*Meriones species*) are widespread [22]. Thus, these ecotones are closely associated with the emergence of ZCL [23]. As the disease is introduced from these ecotones, it is epidemiologically critical to identify the sand fly species involved in this transmission as well as their associated reservoir hosts, their habitats, their respective infection rates with the parasite, and the relationship with the incidence of ZCL.

While several studies performed in Tunisia reported high infection rates of *P. obesus* and *M. shawi* with *L. major* varying from 40 to 70% [6,7], few studies were performed on *L. major* infection prevalence in *P. papatasi* associated with rodent burrows and its association with ZCL. In the present study, we reported that *P. papatasi* is the most abundant sand fly species associated with rodent burrows in all investigated sites. In addition, *L. major* is the only *Leishmania* species infecting *P. papatasi* associated with rodent burrows. Phylogenetic analysis based on a partial region of the ITS-rDNA is in favor of the co-circulation of two different strains, phylogenetically identified as a sub-cluster. Further studies are needed to investigate the epidemiological impact of the circulating *L. major* strains on the incidence of ZCL. The infection prevalence of *P. papatasi* with *L. major* did not differ significantly between the ecotones of *P. obesus* and of *M. shawi*. Our results provide further evidence that *P. papatasi* is indeed the main vector of *L. major* in ZCL endemic areas.

Previous study performed by our group in Hichria and Ouled Mhamed from the delegation of Souk Jedid showed that *P. papatasi* is the main sand fly species associated with rodent burrows in dumpsites and in chenopod field located at the edge of these villages, with an overall minimum infection rate of 5% [24]. Our results are in concordance with studies performed in Iran showing that (1) *P. papatasi* is the most abundant sand fly species associated with burrows of the great gerbil *Rhombomys opimus* [25,26,27,28,29,30], and (2) *L. major* is the most frequent *Leishmania* species circulating among populations of *P. papatasi* associated with rodent burrows.

The infection prevalence of *P. papatasi* associated with burrows of the great gerbil varied from 0.18% [26] to 4% [25]. Similar results were reported in Israel, showing that *P. papatasi* is the most abundant sand fly species (94.4%) associated with burrows of *P. obesus* [23]. In addition, only *L. major* was detected in populations of *P. papatasi* associated with rodent burrows, with an MIR of 2.5% [23]. In Jordan, only *P. papatasi* was associated with burrows of *P. obesus,* and the infection rate with *L. major* was 2% [31].

While the minimum infection rates of *P. papatasi* with *L. major* did not differ significantly between ecotones, ZCL incidence is significantly higher in the *M. shawi* ecotones compared to those of *P. obesus*. This finding could be explained by the high infection prevalence of *M. shawi* with *L. major* (reaching 53% in autumn) [7], and by its migratory behavior leading to the dispersal of ZCL [32]. By comparison, *P. obesus’* sedentary reservoir, living in small colonies as the result of fragmented chenopod fields, maintains a sylvatic cycle of for *L. major*. Considering that the flight range of *P. papatasi* is around 0.75 km [33], increases in densities of *L. major*-infected *P. papatasi* in the ecotone of *M. shawi* expands the overlap of the infected ZCL vector with human habitations and communities, contributing to the emergence of epidemics among naïve human populations.

During the study period, the mean ZCL incidence among delegation was 385.41 ± 77.29. Similar results concerning the period from 1999 to 2004 were reported, showing that the average annual incidence rate of ZCL was 666.7/100,000 inhabitants in the governorate of Sidi Bouzid, and the dynamics of ZCL incidence are significantly heterogenic, occurring in outbreaks and clustering in space and time [11]. Qualitatively, ZCL cases follow the MIR of *P. papatasi* with *L. major* peaks in September–October, as have been reported in Israel [23]. A positive association approaching significance was observed between MIR and the number of ZCL cases. Thus, our results provide strong evidence that MIR can be used in conjunction with other parameters, such as the abundance of the vector and the attack rates, as an integral component of models to assess the risk of ZCL prior to the implementation of effective control measures.

Based on our entomological and epidemiological findings, we provided strong evidence that the two clearly distinct ecotones associated with the interaction of *P. papatasi* with *P. obesus* and *M. shawi* are intertwined and play critical roles regarding the origin of ZCL emergence in rural communities. Our data suggest that for the *P. obesus* ecotone, the balance that exists between rodent and sand fly populations allied with the behavior of this rodent possibly creates a veiled buffer zone for disease emergence. Nevertheless, *L. major*-infected sand flies can still find their way from the burrows of *P. obesus* and transmit ZCL to humans. In contrast, *M. shawi*, due to its invasive and its migratory behavior, likely plays a greater role in disseminating ZCL into the peridomicile [34].

## 5. Conclusions

Breaking the tenuous transmission cycle of *L. major* between reservoir hosts and *P. papatasi* should occur at the ecotone level. Environmental management measures through the destruction of the burrows of *P. obesus* by deep plowing of chenopod fields have at best led to some arguable but generally not sustainable success [11], and with quantifiable adverse environmental effects [11]. In addition, changing chenopod fields to agricultural lands allowed the introduction of *M. shawi*, subsequently exacerbating ZCL transmission. The practice of poisoning rodent reservoir hosts has reduced the incidence of ZCL [35,36], but this approach potentially has high negative impact on the environment. Alternatively, insecticide-treated rodent baits having systemic and feed through insecticidal activity kill blood feeding females and the immature stages that develop and feed on the treated rodent feces, and they have been shown to be effective in controlling sand flies associated with *M. shawi* burrows. However, the impact of insecticide-treated baits on ZCL incidence remains to be determined [8]. From our perspective, joint programs that can bring control methods involving a holistic approach that include management of reservoir hosts and sand flies, together with human behavioral and environmental needs using the One Health approach, are more likely to succeed in reducing the burden of ZCL in North Africa and the Middle East.

## Figures and Tables

**Table 1:** Trapping of sand flies from different habitats.

Trapping Sites (GPS Coordinates)	Ecotone Type (Species of Rodent)	Date	No. of Active Rodent Burrows (Surface of Stick Traps: m^2^)	No. of Trap Nights
Rmilia (009°36′ E, 34°48′ N)	*P. obesus*	09/20 10/09	70/(6 m^2^) 180/(14 m^2^)	420 2550
Awled Mohamed (009°29′ E, 34°52′ N)	*P. obesus*	10/08 10/15	70/(6 m^2^) 55/(4 m^2^)	420 220
Hichria (009°27′ E, 34°53′ N)	*P. obesus*	10/03 10/12	145/(11 m^2^) 150/(12 m^2^)	1595 1800
Khorchef (009°31′ E, 34°44′ N)	*M. shawi*	09/20 10/04 10/14	80/(6 m^2^) 175/(14 m^2^) 150/(12 m^2^)	480 2450 1800
Fayedh (009°36′ E, 34°48′ N)	*P. obesus*	09/24 10/02 10/06 10/21	70/(6 m^2^) 110/(9 m^2^) 205/(16 m^2^) 83/(7 m^2^)	420 990 3280 581
Hinchir Twahria (009°36′ E, 35°04′ N)	*M. shawi*	10/18	105/(8 m^2^)	840
Jabbes (009°36′ E, 34°39′ N)	*M. shawi*	10/23	140/(12 m^2^)	1680
Awled Youssef (009°43′ E, 34°56′ N)	*P. obesus*	10/25	60/(5 m^2^)	300

**Table 2:** Abundance of sand fly species (%) caught in ecotones of *Meriones shawi* and *Psammomys obesus*.

Species	Sex	Biotope		Abundance (%)	
		*M. shawi*	*P. obesus*		
*P. longicuspis*	Female	1	0	1	8 (0.55%)
	Male	3	4	7	
*P. papatasi*	Female	85	332	417	1062 (73.29%)
	Male	171	474	645	
*S. antennata*	Female	1	0	1	16 (1.1%)
	Male	13	2	15	
*S. christophersi*	Female	3	1	4	8 (0.55%)
	Male	3	1	4	
*S. dreyfussi*	Female	0	1	1	1 (0.06%)
	Male	0	0	0	
*S. fallax*	Female	7	18	25	83 (5.72%)
	Male	10	48	58	
*S. minuta*	Female	54	61	115	271 (18.70%)
	Male	111	45	156	
Total	Male	311	574	1449 (100%)	
	Female	151	413		
	Total	462 (31.88%)	987 (68.11%)		

**Table 3:** Minimum infection rates of *P. papatasi* with *L. major* in different biotopes and delegations.

Delegation	Sites	Ecotone	Number of Females Tested	Number of Pools	Number of Positive Pools	MIR
Souk Jedid	Rmilia	*P. obesus*	130	5	3	2.31
	Rmilia	*P. obesus*	66	3	1	1.52
	Hichria	*P. obesus*	75	3	3	4.00
	Hichria	*P. obesus*	30	1	1	3.33
	Awled Mahmed	*P. obesus*	1	1	0	0.00
	Awled Mhamed	*P. obesus*	10	1	1	10.00
Manzel Bouzayen	Korchef	*M. shawi*	6	1	0	0.00
	Korchef	*M. shawi*	13	1	1	7.69
	Korchef	*M. shawi*	4	1	0	0.00
Sidi Bouzid	Fayedh	*P. obesus*	10	1	1	10.00
	Fayedh	*P. obesus*	5	1	0	0.00
	Fayedh	*P. obesus*	3	1	0	0.00
Mazzouna	Hinchir Twahria	*M. shawi*	60	2	0	0.00
Maknassy	Jabbes	*M. shawi*	2	1	0	0.00
Regueb	Awled Youssef	*P. obesus*	2	1	0	0.00
	Total		417	24	11	2.64

**Table 4:** ZCL incidence according to ecotones and delegations.

Delegation	Ecotone	ZCL Cases	Population	Incidence per 100,000 Inhabitants
Souk Jedid	*P. obesus*	68	23,789	286
Manzel Bouzayen	*M. shawi*	187	25,321	739
Sidi Bouzid	*P. obesus*	352	122,670	286
Mazzouna	*M. shawi*	113	24,766	456
Maknassy	*M. shawi*	76	23,789	319
Regueb	*P. obesus*	147	64,988	226

## Data Availability

Not applicable.
